# Clinicopathological and prognostic significance of heat shock proteins in hepatocellular carcinoma: a systematic review and meta-analysis

**DOI:** 10.3389/fonc.2023.1169979

**Published:** 2023-08-04

**Authors:** Dan Xiang, Mengdan Jiang, Ya Chen, Chengjiang Liu, Leilei Li

**Affiliations:** ^1^ Department of Laboratory Medicine, Ya’an People’s Hospital, Yaan, China; ^2^ Department of General Surgery, The First People’s Hospital of Yunnan Province, Kunming, China; ^3^ Department of General Medicine, Affiliated Anqing First People’s Hospital of Anhui Medical University, Anqing, China; ^4^ Disaster Medicine Research Center, West China Hospital, Sichuan University, Chengdu, China

**Keywords:** heat shock proteins, hepatocellular carcinoma, prognosis, clinicopathological significance, meta-analysis

## Abstract

**Background:**

Overexpression of heat shock proteins (HSPs) has been observed in a wide range of human tumors, and there is an increasing evidence demonstrated that HSPs play a key role in tumor progression. Several studies were conducted to explore the clinicopathological characteristics and prognostic value of HSPs in hepatocellular carcinoma (HCC), but the results remain controversial. To address this gap, we conducted a systematic review and meta-analysis.

**Methods:**

The eligible literature was obtained from PubMed, Cochrane library, Web of science, Embase, Chinese National Knowledge Infrastructure and Wan Fang databases. We used the odds ratio (OR) and hazard ratio (HR) as the suitable parameters to assess the clinicopathological features and prognostic value of HSPs in HCC patients.

**Results:**

The meta-analysis results showed that HSPs expression was associated with overall survival (OS) of HCC patients (HR = 1.61, 95%CI = 1.22-2.13, *P*=0.001, *I* ^2 ^= 62.7%). In addition, the pooled results suggested that HSPs expression was significantly correlated with tumor differentiation (OR = 1.33, 95%CI = 1.08-1.65, *P* = 0.907), vascular invasion (OR = 1.31, 95%CI = 1.02-1.69, *P* = 0.921) and lymphatic metastasis (OR=1.98, 95%CI= 1.70-2.31, *P* = 0.740). Meanwhile, the subgroup analysis showed a significant correlation between the expression of HSP27 (HR=1.69, 95%CI = 1.24-2.31, *P* = 0.674) and HSP90α (HR=2.03, 95%CI = 1.73-2.40, *P* = 0.743) with OS of HCC patients.

**Conclusions:**

Our meta-analysis confirms that HSPs expression is closely associated with a worse prognosis in HCC patients, and may be directly involved in tumor differentiation and distant metastasis. In addition, the subgroup analysis results demonstrate that the expression of HSP27 and HSP90α can be served as potential prognostic predictors of HCC.

## Introduction

Primary liver cancer is one of the most aggressive malignant tumors worldwide, with the sixth highest incidence rate and the third highest mortality rate among cancers (1, 2). About 906,000 new cases and more than 830,000 cancer-related deaths were estimated to have occurred in 2020 globally, and hepatocellular carcinoma (HCC) accounts for about 75–85% of all liver cancers (2). Approximately half of all HCC cases and HCC-related deaths occur in China (3). Majority of patients are diagnosed at an advanced stage because of lacking typical clinical symptoms and signs, and less than 20% of HCC patients can undergo surgery for complete resection (4–6). Despite the advances in diagnostic and therapeutic approaches over the past decades, the recurrence rates of HCC patients were high and long-term survival remained disappointed (7–9). The 5-year overall survival of HCC was reported 18% in western countries, and decreased to 12.1% from 20% in China (10, 11). A series of studies have been conducted to explore the molecular and biological mechanism that lead to carcinoma, and researches in identifying novel biomarkers for accurately predicting the clinical features and prognosis of HCC have become a hot topic.

Heat shock proteins (HSPs) are highly conversed molecular chaperones that produced by cells in response to stressful conditions. They are generally classified into five groups based on their molecular weight or systematic gene symbols, including HSP60, HSP70, HSP90, HSP110 and small HSPs (12). This class of proteins were found to be involved in various cellular processes, both physiological and pathological, and profoundly impact tumor progression by manipulating cancer hallmarks such as anti-apoptosis, proliferation, invasion, migration, metastasis, and angiogenesis (13–19). Overexpression of HSPs was observed in a wide range of human malignant tumors, including liver, colorectal, cervical, breast, prostate and lung cancers (20–22). Of particular interest, increasing evidence has demonstrated that HSPs are potential biomarkers for tumor diagnosis, prognosis and therapeutic targets (13). Its prognostic value has been confirmed in various types of solid tumors such as oral, breast and prostate cancers (23–25). Recently, numerous studies attempted to clarify the clinical and prognostic significance of HSPs expression in HCC patients, but their results remained controversial and even paradoxical (26–47). Ji et al. demonstrated that the expression of HSPgp96 was associated with lower tumor, node and metastasis (TNM) stage and favorable prognosis, but they found did not find HSPgp96’s expression association with other clinicopathological features (26). King et al. reported the opposite result, their findings indicated that the expression of HSP27 was significantly related to tumor histology grade (27). In addition, HSP27 expression was found to be significantly related to poor overall survival (OS) (27). Moreover, Kang et al. found that the expression of HSP70 was not correlated with the main clinicopathological features and OS in HCC patients (28). Therefore, we performed this system review and meta-analysis to more precisely investigate the relationships between HSPs expression and the main clinicopathological features of HCC patients. We also evaluated the prognostic value of HSPs expression in patients with HCC.

## Methods

### Literature search

Without any restrictions in terms of publication status, language, and year of publication, all relevant primary studies published on or before May 30, 2023, which assessed the relationship between HSPs expression and the clinicopathological features or prognosis were searched in the following databases: PubMed, Cochrane library, Web of science, Embase, Chinese National Knowledge Infrastructure (CNKI) and Wan Fang databases. The literature search was performed by two researchers independently. The search terms are presented in **Supplementary Table 1** and included the following keywords: heat shock protein and hepatocellular carcinoma. Additional eligible articles in the references list were obtained by manual searching.

### Inclusion and exclusion criteria

Two independent authors screened all relevant articles on the basis of titles and abstracts, and skimmed the full-text according to the inclusion and exclusion criteria. Any disagreement was resolved by discussion with the third author to reach a final consensus. The inclusion criteria of the current system review and meta-analysis were as follows: 1) patients in this study should be clearly diagnosed with HCC; 2) the study was published in Chinese or English, and the full-text is available; 3) the expression of HSPs is measured by immunohistochemistry (IHC) analysis or enzyme-linked immunosorbent assay (ELISA); 4) the article should assess the relationships between HSPs expression and the HCC clinicopathological features or prognosis, and the study include at least one primary outcome of interest; 5) the follow-up periods in assessing prognosis should be more than two years. In addition, the exclusion criteria were applied: letters, reviews, conference abstracts, nonhuman subject studies, case reports, duplicated reports, and data unavailable.

### Data extraction and quality assessment

Two authors independently extracted the following data: first author, publication year, country, HSPs types, sample size, cut-off values, follow-up time, detecting methods, recruitment time, hazard ratios (HRs) or adds ratios (ORs) with 95% confidence intervals (95%CIs) for overall survival (OS) and clinicopathological parameters. The clinicopathological parameters included age(≥60 versus <60), HSPs expression (high/positive versus low/negative), gender (male versus female), tumor differentiation (low versus moderate or high), hepatitis B virus surface antigen (HBsAg) (positive versus negative), alpha-fetoprotein (AFP) (≥ 400ng/ml versus < 400ng/ml), tumor size (≥ 5cm versus < 5cm), vascular invasion (yes versus low), TNM stage (I +II versus III +IV), tumor number (single versus multiple), portal vein tumor thrombus (PVTT) (yes versus no), and lymphatic metastasis (yes versus no).

Newcastle-Ottawa Scale (NOS) was applied to evaluate the quality of original non-randomized studies (48). Three perspectives including selection, comparability and exposure were considered for estimations of the quality and potential bias risks. Studies with a NOS score of ≥6 were considered as a good quality, and ≤ 5 were regarded as a poor quality.

### Statistical analysis

Statistical analysis was performed using the Stata 16.0 software. ORs with 95%CIs were used to evaluate the relationship between HSPs and clinicopathological characteristics in HCC patients. To determine the association between HSPs expression and the prognosis of patients with HCC, HRs with 95% CI were used as the summarized estimates. In case of articles does not provide data directly, the Engauge Digitizer 4.1 software (http://sourceforge.net) was used to extract the survival data from the Kaplan-Meier curves to measure the accuracy of estimated HR according to the guidelines established by Tierney et al. (49). Heterogeneity was estimated using *I^2^* value and Cochran’s Q test. With I^2^>50% or *P*<0.05 suggesting significant heterogeneity (50), the meta-analysis was conducted using a random-effect model. Otherwise, a fixed-effect model was used. To further investigate the source of heterogeneity, we conducted a meta-regression. Sensitivity analysis was performed to determine the stability of aggregated results. In addition, we also assessed potential small-trail effects and publication bias by using Begg’s and Egger’s tests for all available groups with sufficient studies (≥10 studies), and *P*<0.05 indicated the existence of publication bias.

## Results

### Characteristics of included studies

**Figure 1:**
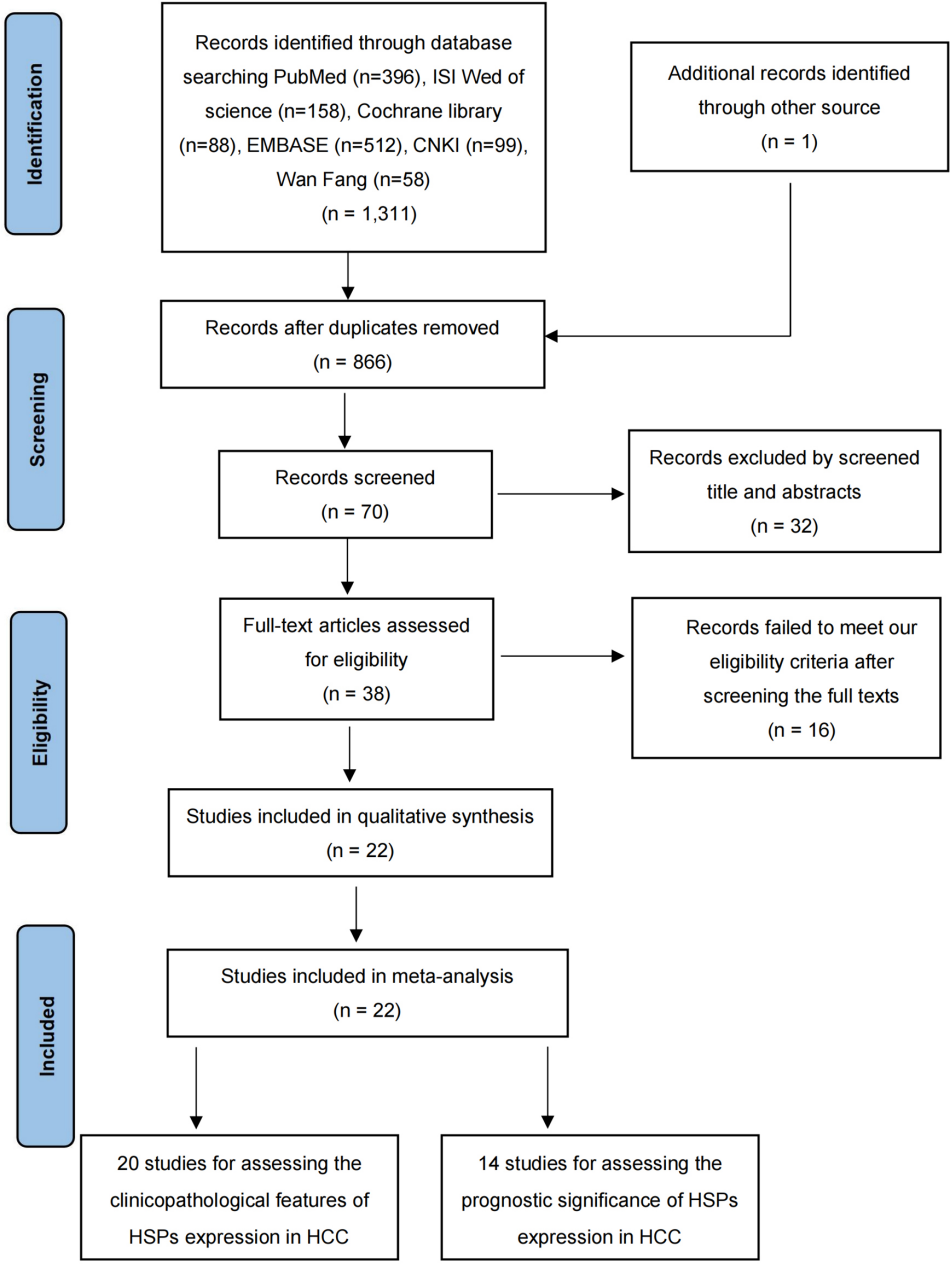
Flow chart outlining the selection of studies.

A total of 1312 records were screened in a primary search for possible related between HSPs expression and the clinicopathological features or prognosis in HCC. **Figure 1** illustrates the process of literature research and selection. Ultimately, 22 eligible studies were included in this meta-analysis (26–47). As shown in **Table 1**, these studies were published from 2000 to 2022, and included a total of 3175 HCC patients. The sample size ranged from 40 to 2150 patients, with 15 of the studies including > 60 patients and 7 of the studies including ≤60 patients. Fourteen studies evaluated patients from China, three from Korea, two from Japan. The NOS score of all these included studies were ≥6, which implied that they were of high quality.

**Table 1:** Characteristics of included studies.

Author	year	Country	HSPs subtype	No.of patients	Cancer type	Detecting methods	Cut-off Value	Investigating fields		Estimates	Recruitment time	NOS Score
								CF	Prognosis			
Ming et al	2016	China	HSP70	110	HCC	IMH	25%	√	√	OR, HR	NR	8
Kang et al	2017	China	HSPA9	49	HCC	IMH	50%	√	√	OR, HR	2006-2010	6
Kang et al	2014	korea	HSP70	83	HCC	IMH	5%	√	√	OR, HR	1999-2011	8
Hung at al	2017	China	HSP27	80	HCC	IMH	NR	√	√	OR, HR	NR	7
Ji et al	2018	China	HSPgp96	84	HCC	IMH	5%	√	√	OR, HR	2007-2009	8
Harimoto et al	2004	Japan	HSP27	60	HCC	IMH	25%	√	√	OR, HR	1991-1998	8
King et al	2000	China	HSP27	58	HCC	IMH	1%	√	√	OR, HR	NR	7
Xu et al	2017	China	HSP90	100	HCC	IMH	10%	√	√	OR, HR	NR	8
Zhang et al	2016	China	HSP27	167	HCC	IMH	25%		√	HR	NR	8
Liu et al	2016b	China	HSP90	60	HCC	IMH	10%	√		OR	2007-2010	7
DAIMEI et al	2016	Japan	HSP27	194	HCC	IMH	NR	√		OR	1993-1997	8
John M et al	2006	China	HSP27	67	HCC	IMH	NR	√		OR	1998-2004	8
			HSP70	67	HCC	IMH	NR	√		OR	1998-2004	8
			GRP78	67	HCC	IMH	NR	√		OR	1998-2004	8
Hua et al	2008	China	HSPgp96	32	HCC	IMH	10%	√		OR	2004-2007	6
Guo et al	2011	China	HSP27	68	HCC	IMH	5%	√		OR	NR	6
Liang et al	2011	China	HSP27	34	HCC	IMH	25%	√		OR	2008-2009	6
Mee et al	2005	korea	HSP27	71	HCC	IMH	10%	√		OR	1996-2003	8
			HSP70	71	HCC	IMH	10%	√		OR	1996-2003	8
Eun et al	2011	korea	HSP70	412	HCC	IMH	NR	√		OR	1995-2004	8
Sun et al	2003	China	HSP90α	40	HCC	IMH	10%	√		OR	1994-2000	6
Dai et al	2009	China	HSP27	45	HCC	IMH	5%	√		OR	2003-2005	6
Chen et al	2022	China	HSP90α	97	HCC	ELISA	135ng/mL	√	√	OR, HR	2019-2020	7
Su et al	2022	China	HSP90α	2150	HCC	ELISA	143.5ng/ml	√	√	OR, HR	2016-2021	8
Zhang et al	2021	China	HSP90α	112	HCC	ELISA	69.3ng/ml		√	OR, HR	2014-2016	8

HCC, hepatocellular carcinoma; CF, clinicopathological features; HSP, heat shock protein; OR, odds ratio; HR, hazard ratio; NR, not reported. "√" represents that the article provides such research data.

### Association between HSPs expression and clinicopathological characteristics in HCC patients

In this study, we evaluated the correlation between HSPs expression and clinicopathological features of patients with HCC, and the results were shown in **Table 2** and **Supplementary Figure 1** , **2**. Fourteen studies, including a total of 1653 HCC patients, were used to evaluate the differential expression of HSPs in HCC and non-HCC tissues. The results revealed that the expression of HSPs in HCC was significantly higher than that in the non-HCC tissues (OR =2.21, 95%CI = 1.50-3.25, *P* = 0.005). The ORs for tumor differentiation were reported in 16 studies that included 1131 cases. The results evaluation of these data indicated that HSPs expression was associated with tumor differentiation (OR = 1.33, 95%CI = 1.08-1.65, *P* = 0.907). Ten studies containing 756 patients showed a significant relationship between HSPs expression and vascular invasion in HCC (OR = 1.31, 95%CI = 1.02-1.69, *P* = 0.921). A greater proportion of patients with high HSPs expression had vascular invasion than those who had low HSPs expression. The ORs for lymphatic metastasis were reported in 6 studies on 2432 patients, and the pooled OR with 95%CI was 1.98 (95%CI = 1.70-2.31, *P* = 0.740), indicating the high HSPs expression was associated with lymphatic metastasis. The pooled results of 16 studies including 3528 patients showed a significant relationship between HSPs expression and gender (OR = 1.19, 95%CI = 1.02-1.39, *P* = 1.000). However, the HSPs expression were not significantly associated with any of the following parameters: HBsAg (OR = 1.03, 95%CI = 0.90-1.18, *P* = 0.907), TNM stage (OR = 1.10, 95%CI = 0.89-1.34, *P* = 0.463), tumor size (OR = 1.18, 95%CI = 0.72-1.96, *P* = 0.000), tumor number (OR = 1.18, 95%CI = 0.77-1.81, *P* = 0.035), AFP (OR = 1.23, 95%CI = 0.57-2.65, *P* = 0.020), PVTT (OR = 1.41, 95%CI = 0.81-2.47, *P* = 0.004).

**Table 2:** Meta analysis of clinicopathological features of heat shock proteins (HSPs) expression in patients with hepatocellular carcinoma (HCC).

Clinicopathological characteristics	N	No. of included patients			Heterogeneity		Model	Pooled OR with 95%CI
		Total	HE	LE	*I*-squared	*P*-value		
Gender (male vs female)	16	3528	1581	1947	0.00%	1.000	Fixed	1.19(1.02-1.39)
Age (≥60 vs <60)	3	259	122	137	0.00%	0.779	Fixed	1.12(0.71-1.78)
Expression (high vs low)	14	1653	935	718	56.40%	0.005	Random	2.21(1.50-3.25)
Differentiation (low vs moderate or high)	16	1131	701	430	0.00%	0.907	Fixed	1.33(1.08-1.65)
HBsAg (posotive vs negative)	14	3403	1510	1893	0.00%	0.907	Fixed	1.03(0.90-1.18)
TNM stage (III+IV vsI+II)	14	1063	666	397	0.00%	0.463	Fixed	1.10(0.89-1.34)
Tumor size (≥5 cm vs < 5cm)	9	2875	1172	1703	82.70%	0.000	Random	1.18(0.72-1.96)
Vascular invasion (yes vs no)	10	756	442	314	0.00%	0.921	Fixed	1.31(1.02-1.69)
Tumor number (multiple vs single)	5	2618	1054	1564	61.40%	0.035	Random	1.18(0.77-1.81)
AFP (≥400ng/ml vs <400ng/ml)	3	2310	849	1461	71.70%	0.020	Random	1.23(0.57-2.65)
Lymphatic metastasis (yes vs no)	6	2432	969	1463	0.00%	0.740	Fixed	1.98 (1.70-2.31)
PVTT (yes vs no)	7	2505	975	1530	68.90%	0.004	Random	1.41(0.81-2.47)

N, number of included studies; HE, positive or high expression; NE, negative or low expression; OR, odds ratio, AFP, alpha-fetoprotein, PVTT, Portal vein tumor thrombus.

### Meta-analysis of OS associated with the expression of HSPs in patients with HCC

**Figure 2:**
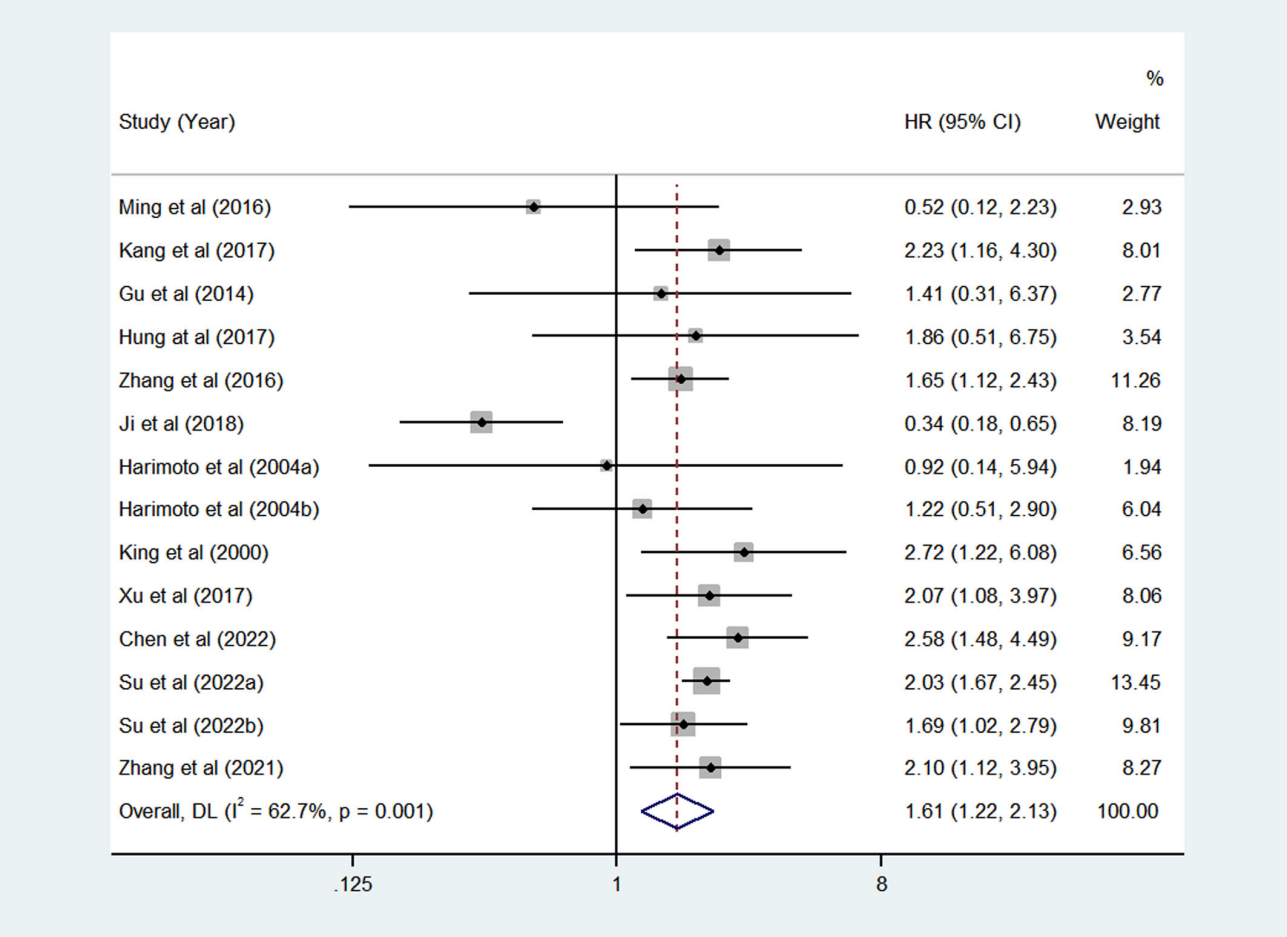
Pooled analyses for assessing the prognostic value of heat shock proteins (HSPs) expression for overall survival (OS) in hepatocellular carcinoma (HCC) patients.

Twelve articles (Harimoto et al. and Su et al. had two set of data) included fourteen studies have reported the relationship between HSPs expression and OS in HCC patients. A total of 2519 HCC patients were included, including 1022 patients with high/positive HSPs expression and 1497 patients with low/negative HSPs expression. As shown in **Figure 2**, the pooled HR of the fourteen studies was 1.61 (95%CI = 1.22-2.13, *P* = 0.001, *I* ^2 ^= 62.7%), indicating significant association betwen HSPs and OS of patients with HCC.

### Subgroup analysis

**Figure 3:**
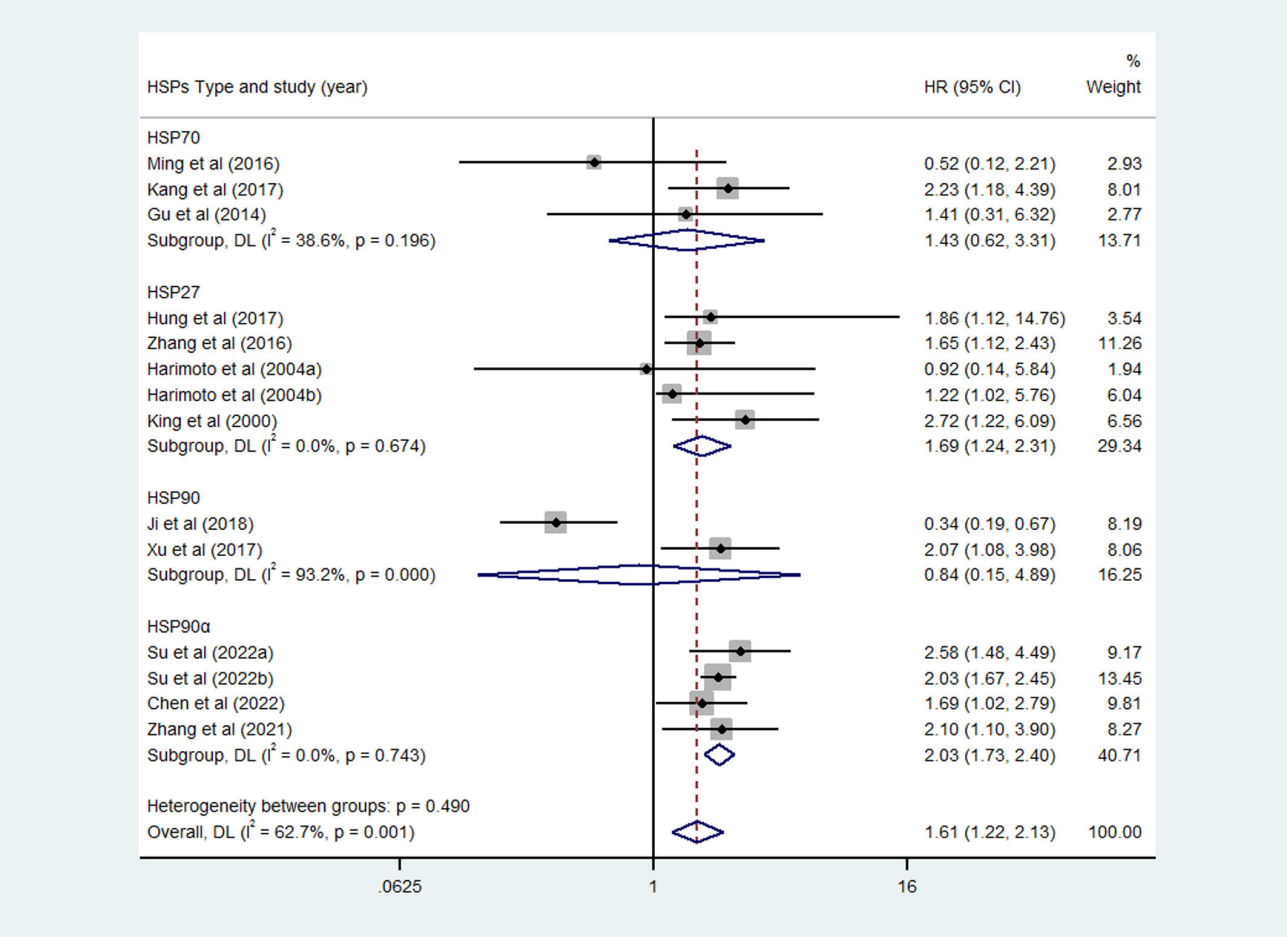
Subgroup analysis of overall survival (OS) of different heat shock proteins (HSPs).

Considering the impact of different HSPs type, we did further analysis. According to the **Figure 3**, the expression of HSP27 and HSP90α were significantly associated with poor OS of HCC, with corresponding pooled HR was 1.69 (95%CI = 1.24-2.31, *P*=0.674) and 2.03 (95%CI = 1.73-2.40, *P*=0.743), respectively. However, the pooled results of HSP70 (HR=1.43, 95%CI = 0.62-3.31, *P*=0.196) and HSP90 (HR=0.84, 95%CI = 0.15-4.89, *P*=0.000) showed that the expression of these HSPs were not significantly associated with OS of HCC patients.

### Meta-regression

To further explore the possible sources of heterogeneity, we conducted a meta-regression based on sample size (≥60, <60), NOS score, country, detecting methods and HSPs type. The results of meta-regression were summarized in **Supplementary Table 2**, and none of the above covariates were found to be a significant source of heterogeneity.

### Publish bias and sensitivity analysis

No publish bias was found for expression, tumor differentiation, HBsAg, TNM stage, and OS, according to the Begg’s and Egger’s test. However, publish bias existed for gender and vascular invasion. The funnel plots obtained from the Begg’s test are shown in **Supplementary Figure 3**, and the corresponding *P* values from Egger’s test are presented in **Table 3**. A sensitivity analysis was also calculated to determine whether individual studies influence the pooled ORs or HRs, and there were no significant impacts on ORs or HRs after excluding any one study, indicating the results were reliability.

**Table 3:** Evaluations for the potential bias within the meta-analysis.

Groups of outcomes	N	Estimates	Egger’s test (P value)	Publication bias
Clinicopathological characteristics				
Gender (male vs female)	16	OR with 95%CI	0.020	Significant
Expression (high vs low)	14	OR with 95%CI	0.340	Not significant
Tumor differentiation (low vs moderate or high)	16	OR with 95%CI	0.760	Not significant
HBsAg (posotive vs negative)	14	OR with 95%CI	0.276	Not significant
TNM stage (III+IV vsI+II)	14	OR with 95%CI	0.332	Not significant
Vascular invasion (yes vs no)	10	OR with 95%CI	0.028	Significant
Prognostic significance				
Overall survival (OS)	14	HR with 95%CI	0.241	Not significant

PVTT, Portal vein tumor thrombus; N, number of included studies.

## Discussion

The role of HSPs in the prognosis and clinicopathological features of HCC has been investigated in several studies, but the results are inconsistent. Therefore, we performed this meta-analysis. The pooled results showed that HSPs expression was associated with poor prognosis in HCC patients (pooled HR = 1.61, 95%CI = 1.22-2.13, *P*=0.001), and the HSPs expression was higher in HCC patients than in normal tissues (pooled HR = 2.21, 95%CI = 1.50-3.25, *P*=0.005). Furthermore, the subgroup analysis confirmed that the expression of HSP27 (pooled HR = 1.69, 95%CI = 1.24-2.31, *P*=0.674) and HSP90α (pooled HR = 2.03, 95%CI = 1.73-2.40, *P*=0.743) can be used as potential prognostic predictors of HCC patients. To further investigate the role of HSPs in HCC, we also analyzed the relationship between HSPs status and clinicopathological features of HCC, the results showed that patients with high expression of HSPs have lower tumor differentiation (pooled OR = 1.33, 95%CI = 1.08-1.65, *P* = 0.907), a higher likelihood of lymphatic metastasis (pooled OR = 1.98, 95%CI = 1.70-2.31, *P* = 0.740), and more prone to vascular invasion (pooled OR = 1.31, 95%CI = 1.02-1.69, *P* = 0.921). Importantly, all of these parameters generally indicate a poor tumor prognosis.

HCC remains a serious challenge to public health due to its high incidence and mortality, and the long-term prognosis of HCC patients is relatively poor. Prognostic markers are widely used in clinical practice and have high clinical value as determinants factors for effective treatment (51). AFP is by far the most widely and extensively studied prognostic marker in HCC. However, the controversy has been raised regarding which specific cut-off for recurrence or survival is selected (52). Moreover, AFP negative tumors account for 30-40% pathologically diagnosed HCC patients, which significantly hampers the application of AFP in HCC prognosis (53–55). Therefore, new prognostic markers are urgently needed.

HSPs were first discovered in 1962 and have since become a hotspot of study due to their ubiquitous presence in all celled organisms (56, 57). Many oncoproteins require high levels of HSPs to maintain their function, and increased levels of HSPs have been found to be significantly higher in tumor cells than that in normal cells, including liver, colorectal, cervical, breast, prostate and lung cancers (20–22). Various molecular mechanisms of HSPs involved in tumor progression and invasion have been investigated over the past few decades, and most of these studies have focused on the expression of HSP27, HSP70 and HSP90 in tumors (17–19, 58–60). HSP27 is an important regulator of the Salvador-Warts-Hippo pathway, which controls tumor inhibition, progression, metastasis, and cancer stem cell programming (13). Eric et al. reported that elevated levels of HSP27 increased the metastatic spread of prostate cancer cells, and confirmed HSP27 as an independent prediction of poor clinical outcome predictor in prostate cancer patients (17). In HCC and lung cancer, HSP70 was reported to promote cancer progression by binding to TLR2 receptor, and inducing MyD88 dependent and independent NF-κB activation and pro-inflammatory gene transcription (18, 58). Furthermore, Kaido et al. reported that HSP70 positively regulates transforming growth factor-α (TGF-α) induced HCC cell migration *via* the AKT signaling pathway (19). In addition, previous studies have demonstrated that the HSP90 expression was associated with tumor proliferation and metastasis (59, 60). HSP90α, a subtype of HSP90, has been proved to participate in induction of tumor cell migration and invasion by binding to LRP1 and activating the ERK and AKT pathways (61). Taken together, HSPs have a significant impact on tumor prognosis and may serve as a predictive or prognostic factor for malignancy.

The most recent meta-analysis in 2018 reported a significant relationship between HSP27 expression and poor prognosis of HCC (62). Hua et al. conducted another meta-analysis on the prognostic role of HSPs in HCC, and the results showed that there was no significant correlation existed between HSPs expression and the prognosis of HCC, but only three eligible articles were included (63). In this study, we included fourteen studies to investigate the prognostic value of HSPs in HCC patients, and finally reached a completely opposite conclusion to the previous meta-analysis (63). Our results confirmed that increased HSPs levels were significantly associated with OS in HCC patients, indicating that HSPs may serve as a potential prognostic predictor of HCC. In addition, we further analyzed the prognostic value of HCC patients among different subtypes of HSPs, including HSP27, HSP70, HSP90 and HSP90α. The pooled results showed that the expression of HSP27 was correlated with poor OS in HCC patients, which was consistent with the previous studies (62). More importantly, our study also confirmed that HSP90α was correlated with poor prognosis of HCC patients, suggesting that HSP90α, like HSP27, was an effective prognostic biomarker for HCC patients. Therefore, HCC patients with increased expression of HSP27 or HSP90α should be advised to shorten the follow-up interval and adjust the treatment plan. A large multicenter clinical trial found that plasma HSP90α decreased after treatment and increased with tumor recurrence (64). Monitoring the expression level of HSPs, especially HSP27 and HSP90α, is a potential approach to predict disease progression and guide in deciding the next treatment strategy. Our findings may also further provide a new insight into the prognosis of HCC patients.

Several limitations should be taken into consideration in this meta-analysis. First, all of the included studies were conducted in Asia population, including China, Korea and Japan, which somewhat limits the universality of our conclusions and may bring bias to draw conclusions about other ethnic groups. Second, there may be a languages bias in this meta-analysis because we only included studies published in English or Chinese. Third, all the included studies were retrospective, which may induce the potential of bias. More high-quality articles are needed to confirm our conclusions. Fourth, the included studies used different detection methods, including IMH and ELISA to test HSPs expression, which may cause some heterogeneity and may also affect the results. Fifth, we used the Kaplan-Meier survival curves to obtain HRs and 95%CIs if the study did not report these data, which may cause bias. Finally, although we conducted meta-regression and sensitivity analysis, the source of heterogeneity could not be fully explained. Overall, the results should be interpreted with caution. More high-quality studies are needed to confirm the results of this meta-analysis.

## Conclusions

In conclusion, our meta-analysis confirms that the expression of HSPs is correlated with poor OS in HCC patients, and may be directly involved in tumor differentiation and distant metastasis. Meanwhile, the subgroup analysis results demonstrate that the expression of HSP27 and HSP90α can severed as effective prognostic predictors of HCC. Additionally, owing to the limitations, more higher quality studies, especially randomized controlled trials, are needed to more precisely validate the clinical role of HSPs in HCC.

## Data availability statement

The original contributions presented in the study are included in the article/**Supplementary Material**. Further inquiries can be directed to the corresponding author.

## Author contributions

All authors contributed to this work. Study design and writing: DX. Constitute figures, tables and data analysis: DX and MJ. Screening of abstracts and articles: MJ, YC and CL. Critically reviewing manuscript: LL. All authors contributed to the article and approved the submitted version.

## Supplementary material

Supplementary Figure 1 Pooled analyses for assessing the associations between heat shock proteins (HSPs) expression and **(A)** Gender, **(B)** Age, **(C)** HSPs expression, **(D)** Differentiation, **(E)** HBsAg, and **(F)** TNM stage.

Supplementary Figure 2 Pooled analyses for assessing the associations between heat shock proteins (HSPs) expression and **(A)** Tumor size, **(B)** Vascular invasion, **(C)** Tumor number, **(D)** alpha-fetoprotein (AFP), **(E)** Lymphatic metastasis, and **(F)** Portal vein tumor thrombus (PVTT).

Supplementary Figure 3 Publication bias for included studies. **(A)** Gender, **(B)** HSPs expression, **(C)** Tumor differentiation, **(D)** HBsAg, **(E)** TNM stage, **(F)** Vascular invasion, and **(G)** Overall survival (OS).
