# Supplementary material for: Clinicopathological and prognostic significance of heat shock proteins in hepatocellular carcinoma: a systematic review and meta-analysis
Source: Front Oncol. 2023 Aug 4;13:1169979. doi: 10.3389/fonc.2023.1169979 (PMC10436519; doi:10.3389/fonc.2023.1169979)
Supplement: Supplementary file 1 [file Table_1.docx]

Supplementary Table 1 Search terms for this network meta-analysis.

| Search number | Query | Results |
| --- | --- | --- |
| 1 | heat shock proteins[Title/Abstract] OR stress proteins[Title/Abstract] OR heat shock protein[Title/Abstract] OR stress protein[Title/Abstract] OR HSPs[Title/Abstract] | 46,592 |
|  |  |  |
|  |  |  |
| 2 | Carcinomas, Hepatocellular[Title/Abstract] OR hepatocellular carcinoma[Title/Abstract] OR hepatocellular cancer[Title/Abstract] OR hepatocellular tumor[Title/Abstract] OR hepatocellular neoplasm[Title/Abstract] OR liver cell carcinoma[Title/Abstract] OR liver cell cancer[Title/Abstract] OR liver cell neoplasm[Title/Abstract] OR liver cell tumor[Title/Abstract] OR HCC[Title/Abstract] | 122,033 |
|  |  |  |
|  |  |  |
|  |  |  |
| 3 | #1 and #2 | 396 |
