# Supplementary material for: Clinicopathological and prognostic significance of heat shock proteins in hepatocellular carcinoma: a systematic review and meta-analysis
Source: Front Oncol. 2023 Aug 4;13:1169979. doi: 10.3389/fonc.2023.1169979 (PMC10436519; doi:10.3389/fonc.2023.1169979)
Supplement: Supplementary file 2 [file Table_2.docx]

Supplementary Table 2 Meta-regression for the potential heterogeneity of overall survival

| Covariates | Exp(b) | SE | t | *P*-value | 95% CI | |
| --- | --- | --- | --- | --- | --- | --- |
|  |  |  |  |  | LCI | HCI |
| Sample size | 0.3040884 | 0.7189832 | 0.42 | 0.683 | -1.35389 | 1.962067 |
| Country | -0.1503742 | 0.4429041 | -0.34 | 0.743 | -1.171713 | 0.8709644 |
| NOS score | 0.2245173 | 0.4412919 | 0.51 | 0.625 | -0.7931035 | 1.242138 |
| HSPs type | -0.1045007 | 0.2241106 | -0.47 | 0.653 | -.6213006 | 0.4122993 |
| Detecting methods | 0.8242206 | 0.6449777 | 1.28 | 0.237 | -.6631006 | 2.311542 |

95% CI, 95% confidence interval.
