# Supplementary figures and images for: Clinicopathological and prognostic significance of heat shock proteins in hepatocellular carcinoma: a systematic review and meta-analysis
Source: Front Oncol. 2023 Aug 4;13:1169979. doi: 10.3389/fonc.2023.1169979 (PMC10436519; doi:10.3389/fonc.2023.1169979)

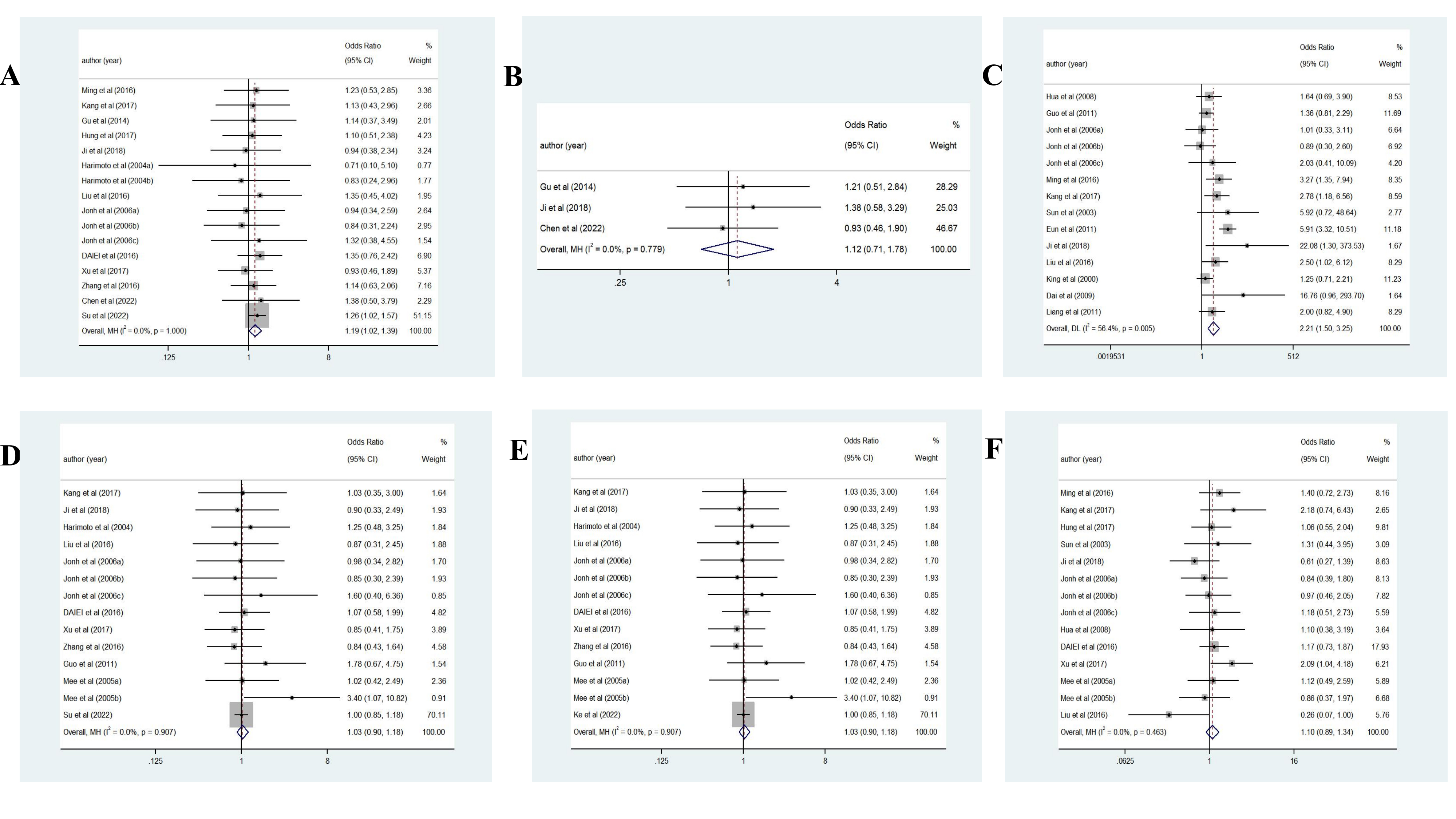

Supplement: Supplementary Figure 1 — Pooled analyses for assessing the associations between heat shock proteins (HSPs) expression and (A) Gender, (B) Age, (C) HSPs expression, (D) Differentiation, (E) HBsAg, and (F) TNM stage. [file Image_1.jpeg]

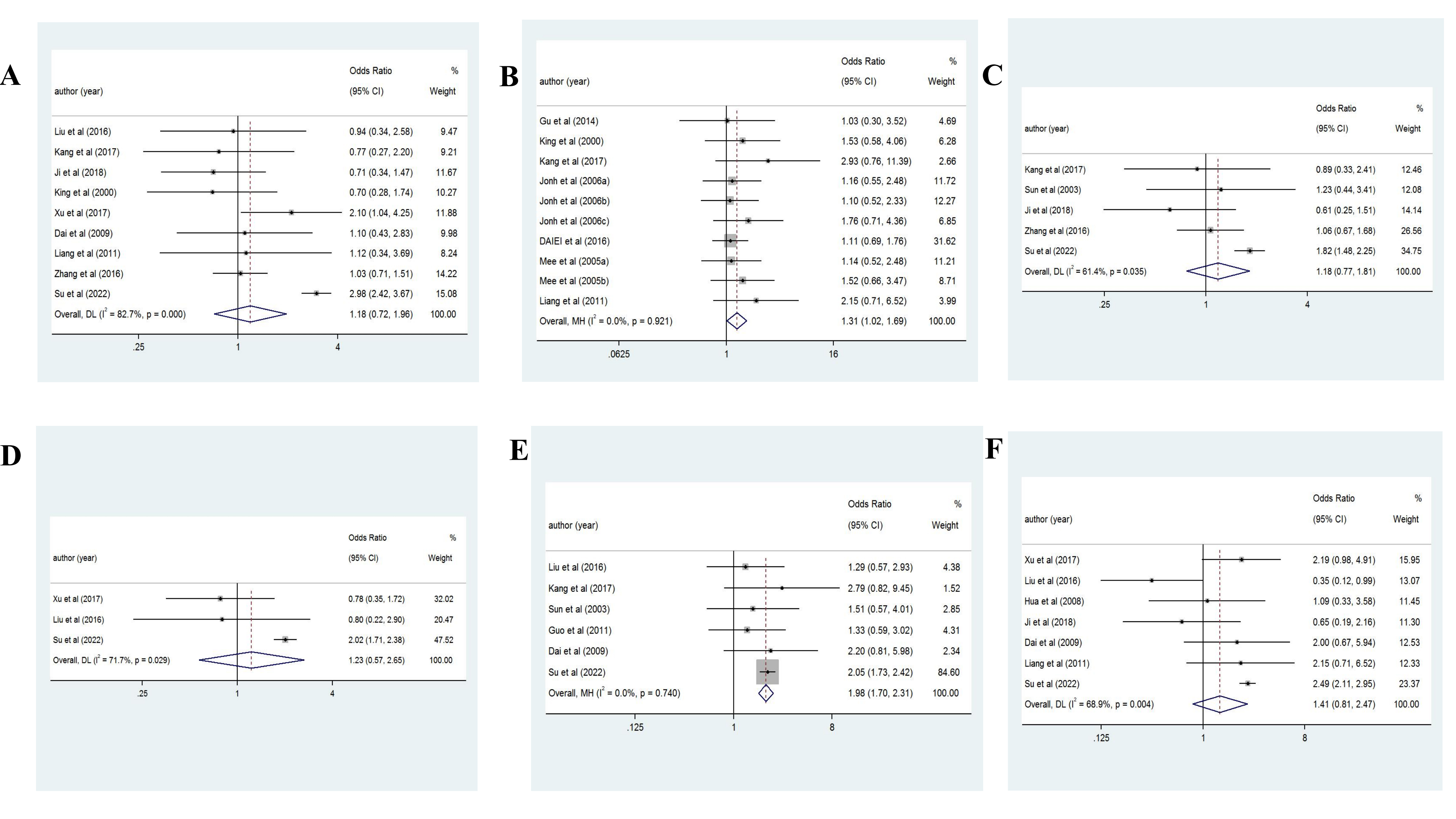

Supplement: Supplementary Figure 2 — Pooled analyses for assessing the associations between heat shock proteins (HSPs) expression and (A) Tumor size, (B) Vascular invasion, (C) Tumor number, (D) alpha-fetoprotein (AFP), (E) Lymphatic metastasis, and (F) Portal vein tumor thrombus (PVTT). [file Image_2.jpeg]

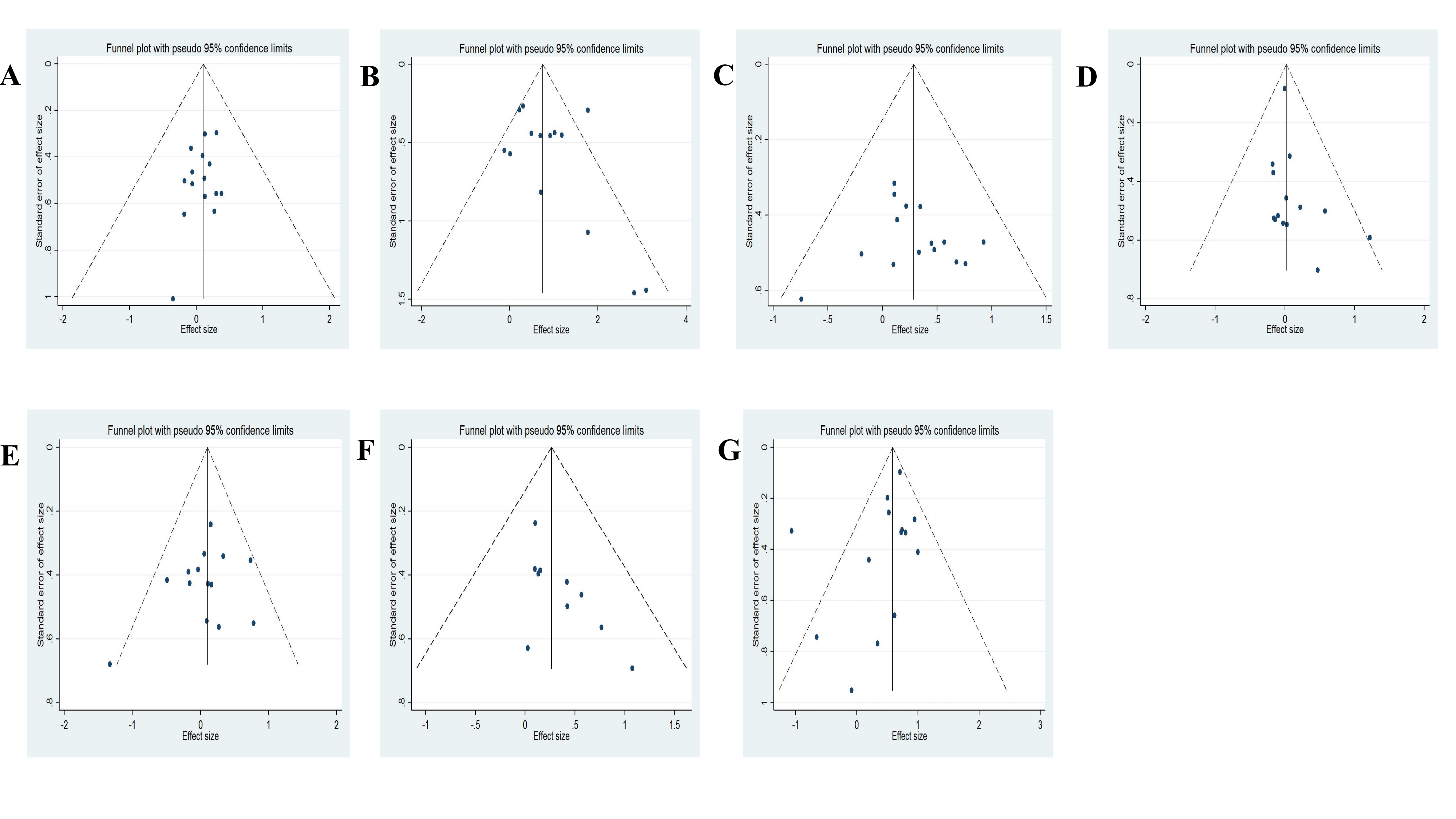

Supplement: Supplementary Figure 3 — Publication bias for included studies. (A) Gender, (B) HSPs expression, (C) Tumor differentiation, (D) HBsAg, (E) TNM stage, (F) Vascular invasion, and (G) Overall survival (OS). [file Image_3.jpeg]
